# The transformative impact of extracellular vesicles on developing sperm

**DOI:** 10.1530/RAF-20-0076

**Published:** 2021-06-25

**Authors:** Michael P Rimmer, Christopher D Gregory, Rod T Mitchell

**Affiliations:** 1 MRC Centre for Reproductive Health, Queens Medical Research Institute, University of Edinburgh, Edinburgh, UK; 2 Centre for Inflammation Research, Queens Medical Research Institute, University of Edinburgh, Edinburgh, UK

**Keywords:** extracellular vesicles, male reproductive tract, prostasomes, epididymosomes, fertility

## Abstract

**Objective:**

To review the role of extracellular vesicles (EVs) released from the male reproductive tract and their impact on developing sperm. We discuss how sperm exiting the seminiferous tubules, although developmentally mature, require further modification. Acquisition of various functions including increased motility, transfer of cargoes and ability to undertake the acrosome reaction is mediated through the interaction between sperm and EVs.

**Methods:**

A review of the literature identified that EVs are released from different portions of the male reproductive tract, notably the epididymis and prostate. These EVs interact with sperm as they pass from the seminiferous tubules to the epididymis and vas deferens prior to ejaculation.

**Results:**

EVs are small lipid-bound particles carrying bespoke RNA, protein and lipid cargoes. These cargoes are loaded based on the state of the parent cell and are used to communicate with recipient cells. In sperm, these cargoes are essential for post-testicular modification.

**Conclusions:**

Interactions between developing sperm and EVs are important for the subsequent function of sperm. Prior to ejaculation, these interactions confer important changes for the post-testicular modification and development of sperm. Little is known about the interaction between EVs from the testes and the spermatogonial stem cell niche or developing sperm within the seminiferous tubules. However, the numerous roles of EVs in the post-testicular modification of sperm have led many to suspect that they may also play important roles in developing sperm within the testes.

**Lay summary:**

Sperm are crucial for successful fertility. In order to do this, they must be able to swim a large distance to meet the egg in the female reproductive tract and fertilise it. Once released from the testes, sperm may appear to be fully developed, but this is not the case. Several important modifications are required in order for them to swim and fertilise an egg. These modifications are carried out by sending sperm small packages from other cells which contain messages and cargo. We discuss the release of these small packages along with different parts of the male reproductive tract and how they change the way sperm behave and function. This article reviews the literature and known functions of these packages called extracellular vesicles, which are released by the male reproductive tract and modify sperm, transforming their function, before they are ejaculated.

## Introduction

### Extracellular vesicles – an overview

Extracellular vesicles (EVs) are a heterogeneous population of small, cell-derived particles, encapsulated by a lipid bilayer. They are secreted by all cell types with wide-ranging functions in both health and disease (Yáñez-Mó *et al.* 2015). Once secreted into the extracellular matrix, they are termed as ‘EVs’ and are unable to replicate (Mause & Weber 2010). Similar to their cell of origin, they express numerous cell surface ligands, in particular, major histocompatibility complex (MHC) molecules (Synowsky *et al.* 2017). The expression of ‘self’ peptides on these MHC molecules makes EVs immunologically inert and, therefore, capable of being widely distributed within the body. Their presence has been reported in all body fluids including blood, urine, cerebrospinal fluid, salvia, breast milk, semen and tears (Pisitkun *et al.* 2004, Keller *et al.* 2007, Mathivanan *et al.* 2010, Baranyai *et al.* 2015, Gui *et al.* 2015, Höög & Lötvall 2015, Riches *et al.* 2016). It is through their widespread distribution and evasion of the immune system which means they are capable of reaching tissues, remote from their cell of origin to mediate their effects. This is perhaps best demonstrated by their role in developing the pre-metastatic niche (Guo *et al.* 2019).

The study of EVs is a rapidly evolving field. First reported in the 1980s, EVs secreted from the reticulocytes of sheep were initially thought to be a means of waste excretion from cells (Johnstone *et al.* 1987). It is now appreciated that this is far from the case, and EVs carry unique and biologically active cargoes including nucleic acids, lipids, proteins and metabolites. Furthermore, loading of these cargoes into EVs is highly ordered, resulting in some being loaded at concentrations several orders of magnitude greater than that of the parent cell (Turchinovich *et al.* 2019, Vasconcelos *et al.* 2019). The precise mechanisms which regulate EV cargo loading are yet to be elucidated. However, it is in part influenced by the health and state of the parent cell (Han *et al.* 2019, O’Neill *et al.* 2019). This variable expression of cargoes implies a highly coordinated process through which cells regulate the messages they transmit to other cells through EVs (Valadi *et al.* 2007).

**Figure 1:**
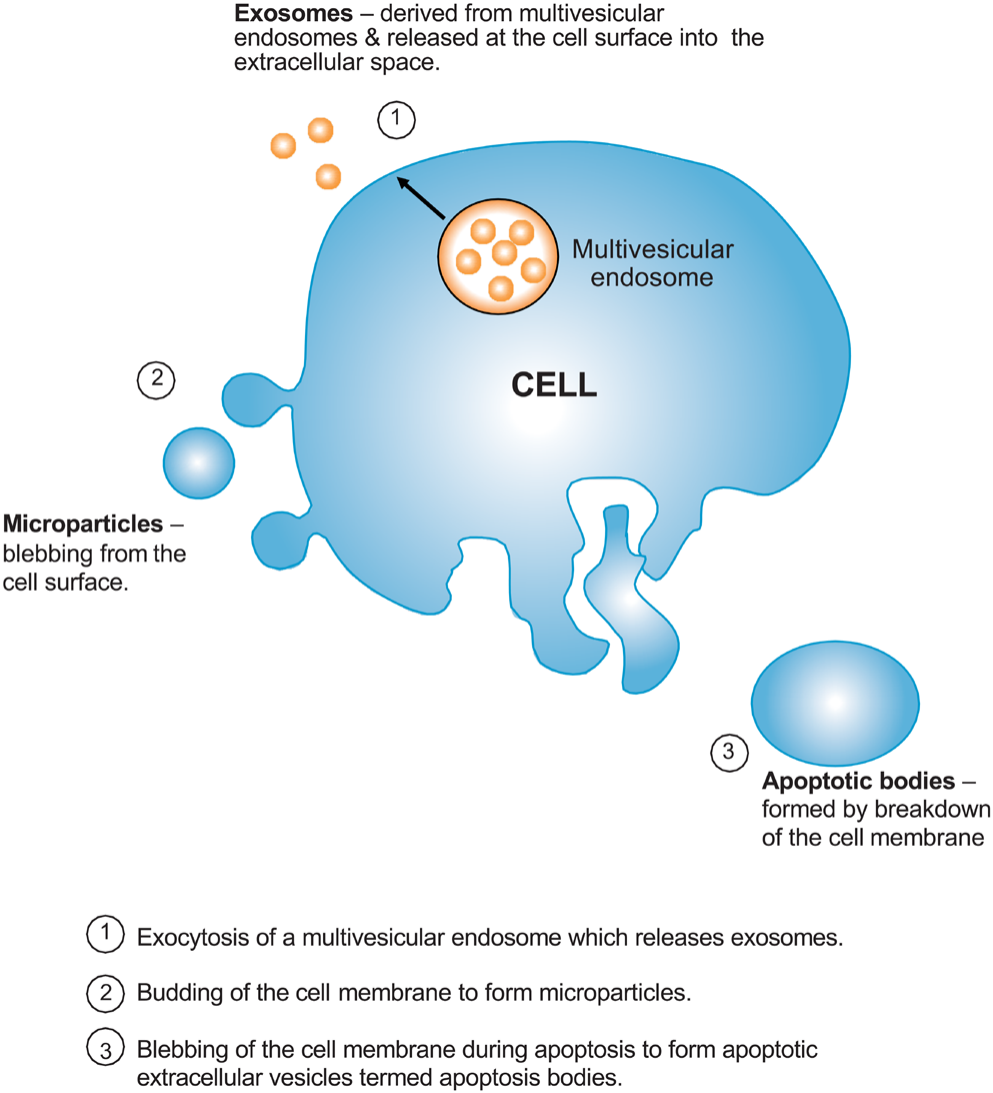
Biogenesis of extracellular vesicles within cells.

**Figure 2:**
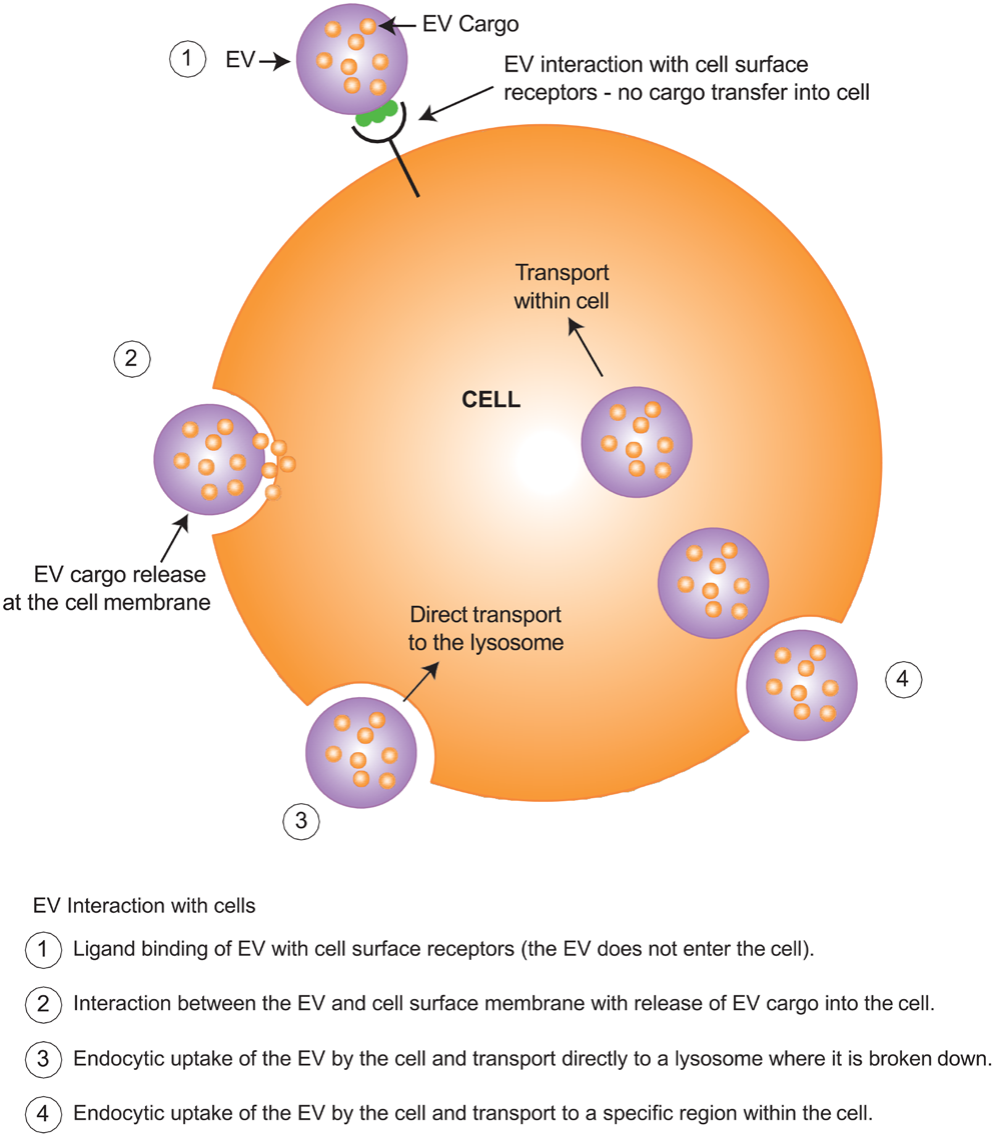
The interaction between EVs with recipient cells.

EVs are extremely diverse in their size, function and biogenesis; it is due to this diversity that attempts to classify them that have often been met with substantial limitations (Witwer & Théry 2019). Discussions regarding their characterisation and classification have resulted in a consensus statement, to outline minimum requirements in studies reporting on EVs (Théry *et al.* 2018). The interaction between cells and EVs broadly occurs through one of four possible pathways: (i) interaction with cell surface ligands; (ii) fusion with the cell membrane and release of cargo into the cell; (iii) endocytic uptake and transport to lysosomes or; (iv) endocytic uptake and transport to specific regions within the cell (Rappa *et al.* 2017), outlined in Figs 1 and 2.

### EVs and the male reproductive tract

Although EVs are now known to have numerous significant physiological functions in many different organ systems, one area in which our understanding of EVs is especially lacking is the development and function of the male reproductive tract. Spermatogonial stem cells within the testis differentiate and mature into sperm within the seminiferous tubules, from puberty and throughout adulthood. As sperm leave the testes and enter the epididymis, they undergo further important changes, many of which are mediated by the uptake of EVs. In particular, EVs from the epididymis and prostate gland interact with sperm during their transition, conferring numerous modifications and gain of function (Martin-DeLeon 2006, Griffiths *et al.* 2008, Park *et al.* 2011, Alasmari *et al.* 2013). Despite the known roles of EVs in the post-pubertal maturation of sperm, the presence and potential role of EVs in the foetal or pre-pubertal testis has yet to be reported.

Here, we review current knowledge of the impact of EVs in the adult male reproductive tract, in particular, their vital role in sperm development and maturation. We highlight how, without the uptake of EVs from the male reproductive tract, aberrant sperm maturation may occur, leading to poor reproductive outcomes. Finally, we consider the unexplored areas in which EVs may play important roles in the male reproductive tract, drawing parallels between other organ systems and physiological roles played by EVs.

### Overview of the development of the adult male reproductive tract

During the 5th week of human embryonic development, the testes develop from bipotential gonads and undergo a series of genotypic and phenotypic changes, involving migration of primordial germ cells and establishment of the germ cell niche (Nef *et al.* 2019). Accessory organs to the reproductive tract such as the prostate, seminal vesicles and urinary tract also develop during this time, forming the urogenital system. During foetal life, the testes descend from the abdominal cavity into the scrotum, through the inguinal canal and provide a highly regulated environment in which developing germ cells mature into functional sperm (Marty *et al.* 2003).

**Figure 3:**
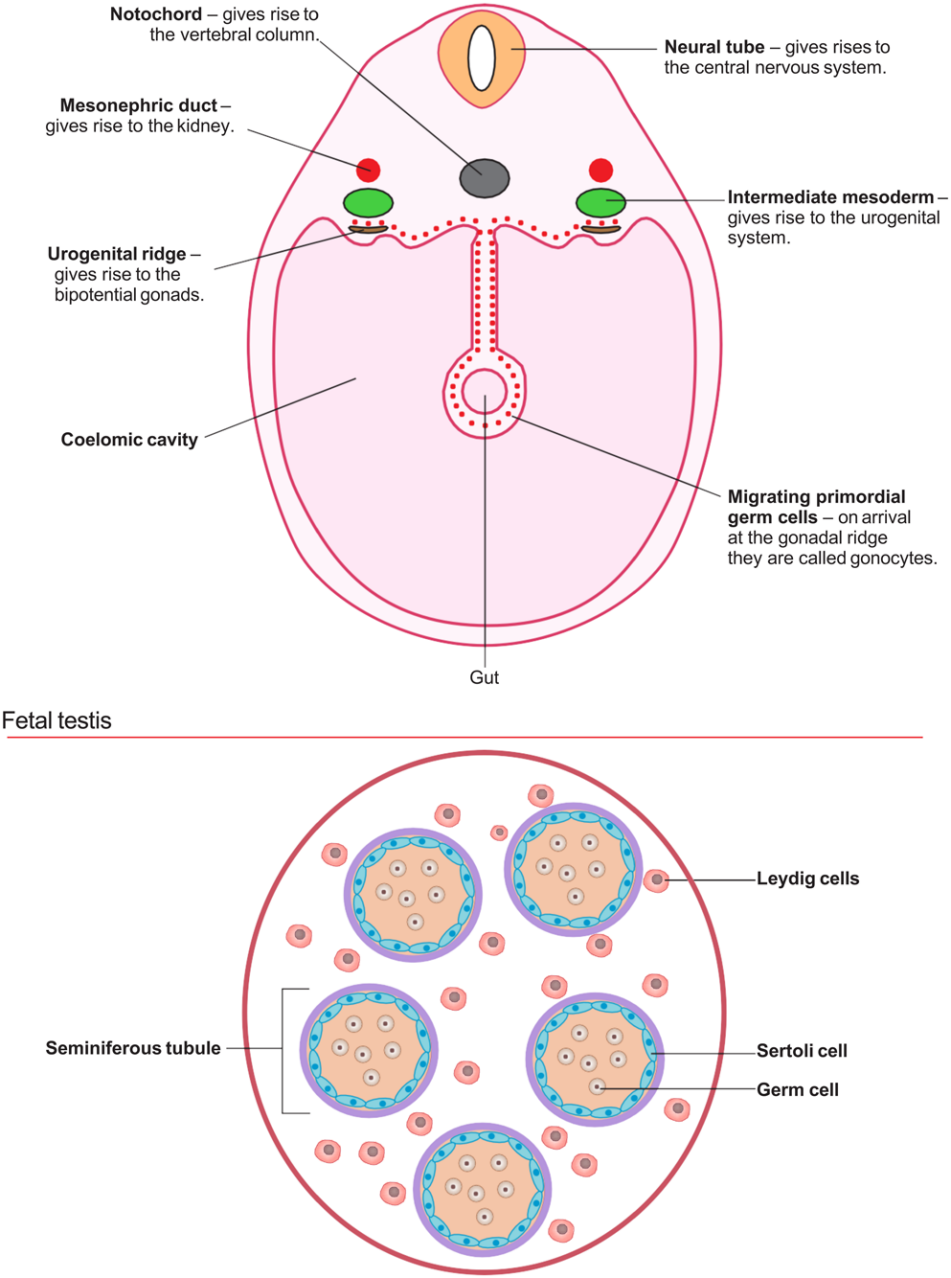
The developing urogenital system: week 4–5.

Normal germ cell maturation and function are critical for future reproductive success. During early embryonic development, primordial germ cells migrate from the coelomic epithelium to the developing testis, outlined in Fig. 3. On their arrival, they are termed as 'gonocytes', the precursors to spermatogonial stem cells. During foetal and early postnatal life, gonocytes undergo a transition into spermatogonia. The transition from gonocyte to spermatogonia establishes a pool of self-replenishing stem cells within the testis. Throughout childhood, these stem cells are self-renewing with a slow turnover compared to their activity following puberty where surges in hormonal stimulation trigger the initiation of spermatogenesis, resulting in a continuous supply of mature, functional sperm (Marty *et al.* 2003).

Pubertal changes in boys typically occur around 11 years of age and are driven by increased activity of the hypothalamic–pituitary–gonadal axis (HPG). Key hormones driving pubertal development include luteinising hormone (LH) which stimulates Leydig cells and follicle-stimulating hormone (FSH) which stimulates Sertoli cells (Marty *et al.* 2003). Both LH and FSH are essential for driving the development of sperm in the testes. The rise in testosterone secreted by LH-stimulated Leydig cells results in the development of secondary sexual characteristics. Within the testis, this rise in testosterone supports spermatogenesis, the differentiation of spermatogonial stem cells into sperm, a continuous process that takes around 74 days.

Spermatogonial stem cell development and subsequently sperm maturation occur in waves, in the highly regulated environment of the seminiferous tubules and spermatogonial stem cell niche (Kostereva & Hofmann 2008). Seminiferous tubules within the testes converge into the rete testis, draining into the efferent ducts of the epididymis (Clement & Giuliano 2015*a*). Despite being morphologically mature, sperm in the rete testes and proximal epididymis are functionally immature and poorly motile.

Within the epididymis, sperm are transformed gaining numerous functions, many of which are mediated by cargo transported to sperm by EVs. These EV-mediated functions include forward motility, zona pellucida binding and the ability to fertilise an oocyte (Martin-DeLeon 2006, Griffiths *et al.* 2008, Sullivan 2016*a*). Following their transit through the epididymis, sperm enter the vas deferens, anastomosing with the prostatic duct and seminal vesicle ducts, prior to ejaculation (Clement & Giuliano 2015*b*).

## Release of extracellular vesicles from the male reproductive tract

### Epididymosomes

Epididymosomes like other EV populations show high levels of heterogeneity, and two distinct sub-populations have been reported in bovine models. The first is smaller and CD9-positive and fuses with sperm via their tetraspanin domains, while the larger is CD9-negative and contains higher levels of epididymal sperm-binding protein 1 (ELSPBP1) (Frenette *et al.* 2010, Caballero *et al.* 2013, Grigor’Eva *et al.* 2017). These EVs are similar in morphology to other EVs of the reproductive tract and are released from the epididymal epithelium through apocrine secretion by the principal cells of the epithelium (Hermo & Jacks 2002, Sullivan & Saez 2013).

The epididymis is a tightly coiled tube on the surface of the testis, conveying sperm from the seminiferous tubules to the ductus deferens, where it joins with the excretory ducts of the seminal vesicles. It has numerous roles including storage, maturation, transport of sperm to the ejaculatory duct and removal of defective sperm. This post-testicular maturation ensures the production of a homogeneous population of sperm. Failure to undergo this transformation produces sperm incapable of fertilising an oocyte (Caballero *et al.* 2010). To mediate these changes, the blood epididymis barrier (BEB) forms a unique and highly regulated microenvironment, shielding the developing sperm from the immune system through tight junctions which limit the passage of immune cells from the blood. A significant number of these changes to sperm function are mediated through the secretion of various molecules from the epididymis, many of these being transported in EVs termed 'epididymosomes' (Sullivan 2016*b*).

Proteomic analysis of human epididymosomes collected during vasectomy identified 146 proteins that differed from that of seminal fluid EVs (Thimon *et al.* 2008). This included enzymes, adhesion, structural and trafficking molecules, but also several with unknown functions. Similar findings were also identified from a bovine model that demonstrated only half the proteins identified in caudal and caput epididymosomes were common to both, in addition to noticeable differences in size (Girouard *et al.* 2011). The cargo of epididymosomes which varies between the epididymal fluid, caudal and distal epididymosomes, is suggestive of differential cargo loading (Girouard *et al.* 2011). Once released into the epididymal fluid, epididymosomes interact with sperm transitioning through the epididymis (Schwarz *et al.* 2013).

Proteomic analysis of mouse epididymosomes also identified segment-to-segment variation; of 1640 proteins identified, 146 were differentially expressed between caput and corpus, while 344 were differentially expressed between corpus and cauda (Nixon *et al.* 2019). Further examination of the protein cargo of epididymosomes in domestic cats, comparing normospermic and teratospermic animals undergoing orchidectomy, identified both common and differentially expressed proteins. EVs isolated from the epididymal caput, corpus and cauda were examined and revealed over 3008 common proteins within EVs related to motility, zona pellucida binding and the acrosome reaction including sorbitol dehydrogenase, hexokinase 1, acrosin, and zona pelucida binding protein (ZPBP) 1 and 2, to name but a few (Rowlison *et al.* 2020). Analysis of the function of these proteins indicated roles in intracellular transport, signalling, metabolism, enzyme and cellular processes, similar to those reported by Gatti *et al.* and Thimon *et al.* Despite similarities between samples, 98 proteins were differentially expressed between normospermic and teratospermic cats, with 7 showing significantly reduced expression in the caudal segment. One protein of particular interest with reduced expression is ZPBP-1, whose function allows sperm to penetrate the zona pellucida and fertilise an ovum. Reduced EV-mediated transfer of ZPBP-1 to sperm may in part lead to reduced fertility associated with teratospermia (Rowlison *et al.* 2020).

Although morphologically ‘normal’, sperm exiting the seminiferous tubules are functionally immature and require further interaction with EVs and uptake of their cargoes. These cargoes are essential for sperm to undergo further modification to undertake their basic physiological function of traversing the female reproductive tract and fertilising an oocyte.

### Role of epididymosomes on sperm maturation

Epididymosomes, like other EVs within the reproductive tract are highly heterogeneous and are important for the development and maturation of sperm. The protein ELSPBP1 was first identified in humans and canines as a sperm-binding protein (SPB) and originates from the epididymis (D’Amours *et al.* 2012*b*). Its structure is similar to the family of SBPs containing four tandemly arranged fibronectin type 2 molecules(Saalmann *et al.* 2001). SBPs bind to sperm following ejaculation and facilitate numerous functions including capacitation and motility (Ekhlasi-Hundrieser *et al.* 2007, Plante *et al.* 2016). In recent years, the presence of ELSPBP1 has been identified in epididymosomes from several other species. Its precise function, however, remains unexplained.

Contents of epididymosomes differ from the luminal fluid in which they are suspended as well as the cells from which they are released (Girouard *et al.* 2011). Interestingly, EVs released by the proximal epididymis carry a different subset of miRNA cargos than EVs released from the distal epididymis (Belleannée *et al.* 2013*a*). The differential expression of >5 or <5-fold change of 83 miRNAs was reported between the epididymal epithelium and epididymosomes, while variation in the miRNA profile between caput and caudally released epididymosomes demonstrated 178 miRNAs with >2 or <2-fold changes in expression (Belleannée *et al.* 2013*a*). Molecules exchanged between epididymosomes and sperm include sperm adhesion molecule 1 (SPAM1) (Martin-DeLeon 2006, Griffiths *et al.* 2008), glioma pathogenesis-related 1-like protein 1 (GliPr1L1) (Caballero *et al.* 2012) and metalloproteases (Oh *et al.* 2009); all of which play a role in fertilisation. Numerous other proteins transferred by epididymosomes to sperm appear to have roles including capacitation and motility such as the proto-oncogene tyrosine-protein kinase Src (cSrc) and macrophage migration inhibitory factor (MIF) (Frenette *et al.* 2002, Eickhoff *et al.* 2004, Krapf *et al.* 2012). Protection of sperm DNA from oxidative stress is conveyed through the transfer of non-conventional glutathione peroxidase 5 (GPX5) (Chabory *et al.* 2009), while the transfer of Liprin α3 is an important component for sperm to undergo the acrosome reaction (Gervasi & Visconti 2017). Despite the extensive understanding of some cargos delivered to sperm through epididymosomes, not all have a clearly defined function, for example,methylmalonate-semialdehyde dehydrogenase (MMSDH) (Suryawanshi *et al.* 2012).

Evidence supporting the role of epididymosomes in sperm maturation can be drawn from the changing morphology and cargo of epididymosomes released from different segments of the epididymal lumen as well as changes within sperm (Trigg *et al.* 2019). This is correlated with sperm acquisition of function including motility and capacity to fertilise an ovum (Sullivan 2016*a*). Epididymosomal cargo incorporated into sperm is transported to specific areas within the sperm based on the region of the epididymis from which they are secreted (Sullivan *et al.* 2007, Nixon *et al.* 2019). These molecules have roles in sperm motility such as sperm migration inhibitory factor which is transported to the fibres of the flagellum (Eickhoff *et al.* 2001, Eickhoff *et al.* 2006). Molecules regulating binding to the zona pellucida, for example, P26h/P25b, are transported to the plasma membrane, overlying the acrosome (Légaré *et al.* 1999).

Although the specific role of epididymosome miRNA on future offspring in humans is unclear, it is well established that miRNAs within epididymosomes released by the proximal epididymis alter the gene expression of epididymal cells in the distal portion (Belleannée *et al.* 2012, Belleannée *et al.* 2013*b*). The impact this has on subsequent epididymosome release and their cargo is yet to be determined.

Proteomic analysis from a bovine model demonstrated that only half the proteins identified in caudal and caput epididymosomes were common to both, in addition to notable differences in size (Girouard *et al.* 2011). The cargo of epididymosomes which varies between the epididymal fluid, caudal and distal epididymosomes, is suggestive of differential cargo loading (Girouard *et al.* 2011). Once released into the epididymal fluid, epididymosomes interact with sperm transitioning through the epididymis (Schwarz *et al.* 2013).

### Elimination of defective sperm

Although the precise function of ELSPBP1 within EVs is unknown, its presence within sperm is the characteristic of sperm that have died, prior to ejaculation. It has been identified in sub-fertile bulls, and further analysis has shown it to be present only in the dead sperm (D’Amours *et al.* 2012*a*). Although the expression of ELSPBP1 can be used to characterise dead sperm within the epididymis, its presence was only identified within a subset of dead sperm in the proximal epididymis, but all dead sperm in the distal epididymis (D’Amours *et al.* 2012*b*). This finding is suggestive of secretion by the epididymis and uptake of ELSPBP1 by sperm as they transit through the epididymis. Discussions as to its possible function have led some to speculate that its presence within dead sperm may be protective to live sperm (D’Amours *et al.* 2012*b*), similar to fibrinogen-related protein (FGL2), another epididymal protein associated with non-viable sperm (Olson *et al.* 2004). FGL2 forms a protective coat around non-viable sperm, shielding viable sperm from the release of deleterious enzymes (D’Amours *et al.* 2012*b*).

### EV-mediated changes to offspring

Modification of RNA within sperm, through external stimuli, can alter paternally inherited characteristics in the offspring such as insulin sensitivity (Gapp *et al.* 2014, Chen *et al.* 2016), although the ability for epididymal EV-mediated RNA to confer these epigenetic modifications was only recently proven (Maciel & Mansuy 2019). Chan *et al.* identified that paternal environmental stressors were conveyed to developing sperm by epididymal EVs, mediating post-testicular germline modifications to sperm. This was further studied in an *in vitro* model using corticosterone to mimic physiological stress, which altered miRNA and protein cargoes of EVs. Incubation of these EVs with sperm and subsequent intracytoplasmic sperm injection to oocytes led to transcriptomic alterations in both placental and brain tissue in developing mouse embryos (Chan *et al.* 2020).

At present, the specific role of epididymosomal cargoes on future offspring in humans is unclear. The ability for proximal epididymal miRNA cargoes to alter gene expression on the distal epididymis is well established and highlights the potential for these molecules to have, as yet, unidentified impacts on the offspring (Belleannée *et al.* 2012, Belleannée *et al.* 2013*b*).

### Prostasomes and seminal fluid extracellular vesicles

The prostate gland is the largest of the male reproductive accessory organs and sits at the base of the urinary bladder. Secretions from the prostatic epithelium into the prostatic ducts make up to one-sixth of the ejaculate which then mixes with sperm from the vas deferens. The identification of EV-like storage vesicles within the prostate, termed 'prostasomes', provides tentative evidence of their generation from a similar biogenesis pathway to that of exosomes, formed within the multivesicular endosome of the cell.

Prostasomes were first reported by Ronquist *et al.* in 1978 and described as submicron membrane-bound secretory granules released by the acinar cells of the prostate gland (Ronquist *et al.* 1978*a*,*b*). They form a part of the numerous fractions of EVs within semen as they mix with those from the epididymis and other areas of the reproductive tract (Höög & Lötvall 2015, Jodar *et al.* 2017).

Like other EV populations, prostasomes are heterogeneous, ranging in size from 40–500 nm (Ronquist *et al.* 1978*a*,*b*). Their characterisation has led to the identification of two distinct sub-populations based on morphology; one larger cohort and one smaller, more electron-dense cohort. The formation of prostasomes appears to differ from that of epididymosomes. Their assembly has been visualised within the apical region of the cell, where the golgi apparatus is most abundant. The size profile of prostasomes is similar to storage vesicles within the prostate epithelium, leading to speculation that these storage vesicles may be prostasomes yet to be released or share a similar biogenesis pathway (Ronquist & Brody 1985). Further imaging has identified similarities between storage vesicle production within the prostate and multivesicular bodies, a site of EV production within cells (Sahlén *et al.* 2002). Surface markers of prostasomes, albeit produced from a prostate cancer cell line (PC-3), are enriched with several multivesicular bodies and EV markers including hepatocyte growth factor-regulated tyrosine kinase substrate (HGS), lysosomal-associated membrane protein 1 and 2 (LAMP1 and LAMP2), and tumour susceptibility gene 101 (TSG101) (Llorente *et al.* 2004, Llorente *et al.* 2007). Despite similarities between prostasomes and prostate storage vesicles, it has never been conclusively demonstrated that storage vesicles observed within the prostate are the precursors to prostasomes released into the extracellular environment.

### Role of prostasomes on sperm maturation

Prior to the interaction between sperm and prostasomes or seminal fluid EVs, sperm undergo differentiation and maturation within the highly regulated and immune-privileged environment of the testes. This is essential for their development. Following ejaculation, however, further modifications are required for the successful fertilisation of an oocyte, many of which are mediated through prostasomes.

The interaction of EVs with sperm following ejaculation and transfer of Ca^2+^ highlights one of their many roles in post-testicular modification to sperm. Although sperm used in intracytoplasmic sperm injection techniques are extracted from the epididymis and capable of fertilising an oocyte, they are artificially introduced to the cytoplasm. This bypasses the need to traverse the female reproductive tract and carry out the acrosome reaction, both essential prerequisites for successful fertilisation. Examination of the EVs present within semen revealed a diverse mixture of individual vesicles but also multifaceted structures which tentatively points to the release of EVs from other portions of the reproductive tract which are yet to be characterised (Höög & Lötvall 2015). This heterogeneity of EVs within the seminal fluid, the origins of which may be poorly characterised, means the function of these EVs may not be exclusively attributed to a subset of known EVs, such as prostasomes.

Since their discovery, the potential functions of prostasomes and seminal fluid EVs have been widely investigated. Sperm ejaculated into the female reproductive tract, although developmentally mature, still needs to undergo further modification to acquire fertilising potential (Bailey 2010). These processes are collectedly termed as 'capacitation' and include zona pellucida binding, the acrosome reaction and the potential to interact with the oocyte plasma membrane. Unsurprisingly, several seminal fluid EV functions have been identified, the most significant of which is their ability to directly fuse with sperm, similar to epididymosomes (Kravets *et al.* 2000). Unlike epididymosomes, however, the interaction between sperm and seminal fluid EVs appears to occur following ejaculation as they interact in the female reproductive tract and are discussed in more detail below (Aalberts *et al.* 2014).

Analysis of the composition of seminal fluid EVs comparing asthenozoospermic and normozoospermic patients identified the differential expression of the protein transient receptor potential vanilloid subfamily member 6 (TRPV6) (Lin *et al.* 2019). TRPV6 is an epithelial Ca^2+^ channel, regulating intraluminal Ca^2+^, and knock-out of the TRPV6 gene has been shown to disrupt Ca^2+^ absorption within the epididymal epithelium resulting in higher intraluminal Ca^2+^ (Weissgerber *et al.* 2012). In addition to disrupted Ca^2+^ regulation, decreased levels of TRPV6 within seminal fluid EVs and spermatozoa were associated with asthenozoospermia and reduced fertilization capacity (Weissgerber *et al.* 2012, Lin *et al.* 2019). Given our understanding of the importance of Ca^2+^ regulation in sperm motility, lower levels of TRPV6 within EVs and reduced EV-mediated transfer of TRPV6 may play an important role in conferring motility to developing sperm.

### Extracellular vesicles and male gamete function

The interaction of sperm and seminal fluid EVs occurring only after ejaculation means the majority of their association and transfer of cargo occurs in the lower female reproductive tract (Arienti *et al.* 2004). This may be why interactions between sperm and seminal fluid EVs favour a slightly acidic pH, as demonstrated in *in vitro* studies using equine sperm (Aalberts *et al.* 2013), an environment similar to the female reproductive tract. Interestingly, these interactions vary based on pH, with EVs binding to the mid-piece of sperm in an acidic environment but the head of sperm in a neutral pH (Arienti *et al.* 1997, Park *et al.* 2011). This interaction is associated with increased sperm survival in the lower female genital tract and enables sperm to pass through the cervical mucus and traverse the great distance to the fallopian tubes to fertilise on oocyte (Ronquist & Nilsson 2004). Sperm within the ejaculate are not yet fully functional and must first undergo capacitation (Fraser 2010). The abundance of protamines within sperm which replace histones during spermatogenesis means they are unable to synthesise proteins and rely on interaction with the external environment for further modification, such as interaction with EVs (Samanta *et al.* 2016).

Capacitation refers to a process which describes the functional maturation of sperm. These changes include increased motility and potential to bind to the zona pellucida via receptors on the sperm head (Aalberts *et al.* 2014). These processes are driven by protein kinase C activity within sperm and are stimulated by cAMP. Delivery of cAMP within prostasomes leads to higher levels of cAMP within sperm (Fraser 2010).

In addition to cAMP and cargoes to undertake capacitation, prostasomes deliver other essential cargo to sperm, notably cyclic adenosine diphosphoribose and Ca^2+^ signalling machinery (Park *et al.* 2011). Ca^2+^ regulation within sperm is essential for its motility and interaction with the oocyte when carrying out the acrosome reaction (Park *et al.* 2011, Alasmari *et al.* 2013). Despite the transfer of epidydimal cargoes such as ADAM-7, MIF and aldose reductase which promote motility, prostasomes transfer their own unique cargoes to further promote sperm motility.

The impact of prostasomes on sperm is dynamic (Aalberts *et al.* 2014). Despite evidence suggesting the interaction between sperm and prostasomes increase the activity of sperm, prostasomes also confer inhibitory changes. In particular, a reduction in levels of tyrosine phosphorylation negatively impacts capacitation; however, this has only been demonstrated in one study (Pons-Rejraji *et al.* 2011). Prostasomes are also reported to reduce motility through the delivery of Zn^2+^ ions. The presumption that Zn^2+^ resides within prostasomes is supported by lower levels of Zn^2+^ in prostasome-free semen samples. However, this observation does not definitively confirm the presence of Zn^2^ within prostasomes (Vivacqua *et al.* 2004).

Interestingly, the cargo delivered by prostasomes to sperm is predominantly to the neckpiece of the sperm, as opposed to the head and suggests EVs are the targeted specific regions of the sperm (Park *et al.* 2011). It should be noted that altered prostasome binding to different regions on the sperm may be due to expression of alternative ligands by the sperm and not due to changes to the binding potential of the prostasome.

As well as their interactions with EVs from the male reproductive tract,* in vitro* models have demonstrated uptake of endometrial EVs by spermatozoa (Murdica *et al.* 2020). Fluorescently labelled EVs derived from cultured endometrial cells in the proliferative phase demonstrated an increased uptake by sperm compared to their secretory phase counterparts. Uptake of secretory phase EVs was then shown in increased capacitation potential within sperm and likely reflects one of the many dynamic interplays between the male gamete and female reproductive tract in preparation for fertilisation and subsequent implantation of an embryo (Martin-DeLeon 2016, Murdica *et al.* 2020).

### Proteomic analysis

Proteomic analysis by Utleg *et al.* identified over 139 proteins using mass spectrometry of prostasome cargoes. However, these were obtained from EVs within seminal fluid and may represent a heterogeneous EV population. Some were specific to the prostate, such as prostate-specific antigen (PSA) and prostatic acid phosphatase (PAP), but many had not been previously identified within the seminal fluid EVs (Utleg *et al.* 2003). Analysis of these proteins’ functions demonstrated that the most abundant group was enzymes, followed by transport and structural proteins. These findings suggest prostasome cargo may have the potential to influence a cell’s metabolic state. Additional functions of seminal fluid EVs contents include PSA and peptide hydrolases which liquefy semen and degrade seminal proteins, respectively (Utleg *et al.* 2003). Structural and transport proteins such as annexins, actin and profilin, which have important functions in calcium-binding, cell structure and actin-binding have also been identified (Utleg *et al.* 2003). Other molecules within seminal fluid EVs include G-binding, chaperone and signal transduction proteins (Utleg *et al.* 2003, Burden *et al.* 2006).

Although 684 proteins were identified in both cohorts of EVs, 539 were exclusively detected within large EVs and 7 in small EVs. Assessment of the functions of these proteins revealed distinct molecular functions between large and small EVs including cell growth, apoptosis, cell communication, metabolism and signal transduction (Zhang *et al.* 2020). A similar study by Yang *et al.* also reported numerous proteins within seminal plasma EVs characterised by mass spectrometry. To be confident that the proteins identified originated from EVs, both sperm cell lysate and EV depleted seminal plasma were used as controls. Molecular and biological functions identified by gene enrichment analysis include catalytic activity, GTPase activity, metabolism and cell growth and maintenance (Yang *et al.* 2017).

Although a heterogeneous source of EVs, proteomic analysis of seminal fluid EVs was undertaken by Lin *et al.* comparing asthenozoospermic and normozoospermic patients. Differential expression of 11 upregulated and 80 downregulated proteins was identified in asthenozoospermic patients from 3699 uniquely identified proteins. Gene ontology analysis of these proteins demonstrated roles in transcription, proteolysis, metabolism, protein binding, intracellular transport and autophagy (Lin *et al.* 2019). Impaired ability of sperm to carry out these essential roles clearly demonstrates how sperm with impaired cargo transfer from EVs will fail to develop fully, and patients identified clinically as asthenozoospermic.

As discussed earlier in this review, the proteomic analysis of human epididymosomes obtained during a vasectomy identified 146 individual proteins which differed from proteins identified within seminal fluid EVs (Thimon *et al.* 2008). These proteins had numerous functions which included enzymes, adhesion, structural and trafficking molecules, but also several whose functions have not yet been characterised. Despite the numerous functions epididymosomes play in sperm maturation, for several of these proteins it has yet to be elucidated if they are transferred to sperm, or lead to their modification in any meaningful way.

Due to procedural limitations in this study, in obtaining epididymosomes specifically from the patients’ caput, corpus and caudal epididymis, Thimon *et al.* compared mRNAs identified from their patient cohort to known mRNAs from published datasets. mRNAs from 19 proteins were differentially expressed and were suggestive of EVs secreted predominantly from the caput (*n* = 13) but also the corpus (*n* = 3) and cauda (*n* = 3) of the epididymis (Thimon *et al.* 2008).

A similar study by Gatti *et al.* also studied the protein composition of epididymosomes in rams identifying the distinct differences between EVs obtained from caudal fluid, seminal plasma and sperm extracts. Numerous functions of these proteins were reported, as with the study by Thimon *et al.*, including transport proteins, enzymes, cytoskeletal proteins and vesicle-associated proteins (Gatti *et al.* 2005). Analysis of EVs from different segments of the epididymis identified not all were secreted equally with proteins such as phosphodiesterase E-NPP3 predominantly secreted from the caudal epithelium (Gatti *et al.* 2005).

### EV interaction with the immune system

The interaction between EVs and the immune system is widely characterised, with an overview of several of these interactions both in the male and female reproductive tract discussed below (Poon *et al.* 2018, Mittal *et al.* 2020, Zhou *et al.* 2020).

Of the many interactions within the male reproductive tract, of particular interest is CD47 expressed both by large prostate EVs and also by tumour cells. CD47 modulates the immune system and acts as an anti-phagocytic signal to macrophages (Chao *et al.* 2019). EV-mediated transfer of CD47 to sperm may also confer some protection from macrophage phagocytosis within the female reproductive tract (Zhang *et al.* 2020). This protection through the impaired immune response of macrophages may be one of the many roles seminal fluid plays in the female reproductive tract to allow sperm to survive to fertilise an oocyte. Further EV interactions with the immune system have been identified by prostasomes, a component of seminal fluid EVs which both impaired lymphocyte proliferation and impaired neutrophil functions (Skibinski *et al.* 1992).

Modulation of the immune response by EVs within seminal fluid continues once sperm enter the female reproductive tract and plays several roles in adapting the maternal immune response both to sperm and the developing semi-allogenic conceptus (Tannetta *et al.* 2014). Interaction between CD48 expressing prostasomes and uterine natural killer (uNK) cells showed both a reduction in the uNK activating receptor CD244 but also reduced uNK activity and expression of interferon-gamma (Tarazona *et al.* 2011). The combination of these effects may be protective to sperm traversing the female reproductive tract and subsequent embryo implantation. In addition to CD48 expression, prostasomes expressed CD59 and were protective to sperm from the complement system of the female reproductive tract (Rooney *et al.* 1993). In a porcine model, the interaction between seminal fluid EVs and the endometrium demonstrated EV-mediated alteration to the transcriptome of both immunoregulatory and inflammatory pathways (Bai *et al.* 2018). In particular, downregulated T cell differentiation is suggestive of EV-mediated protection to sperm within the female reproductive tract, while changes to inflammatory pathways may be a crucial aspect of the highly regulated window of embryo implantation (Ochoa-Bernal & Fazleabas 2020). Although seminal fluid, for which EVs are a component, mediates changes in both gene expression and inflammation, the impact of these changes has not been clearly attributed to EVs (Robertson *et al.* 2009, Sharkey *et al.* 2012).

### Lesser characterised EVs of the male reproductive tract

**Figure 4:**
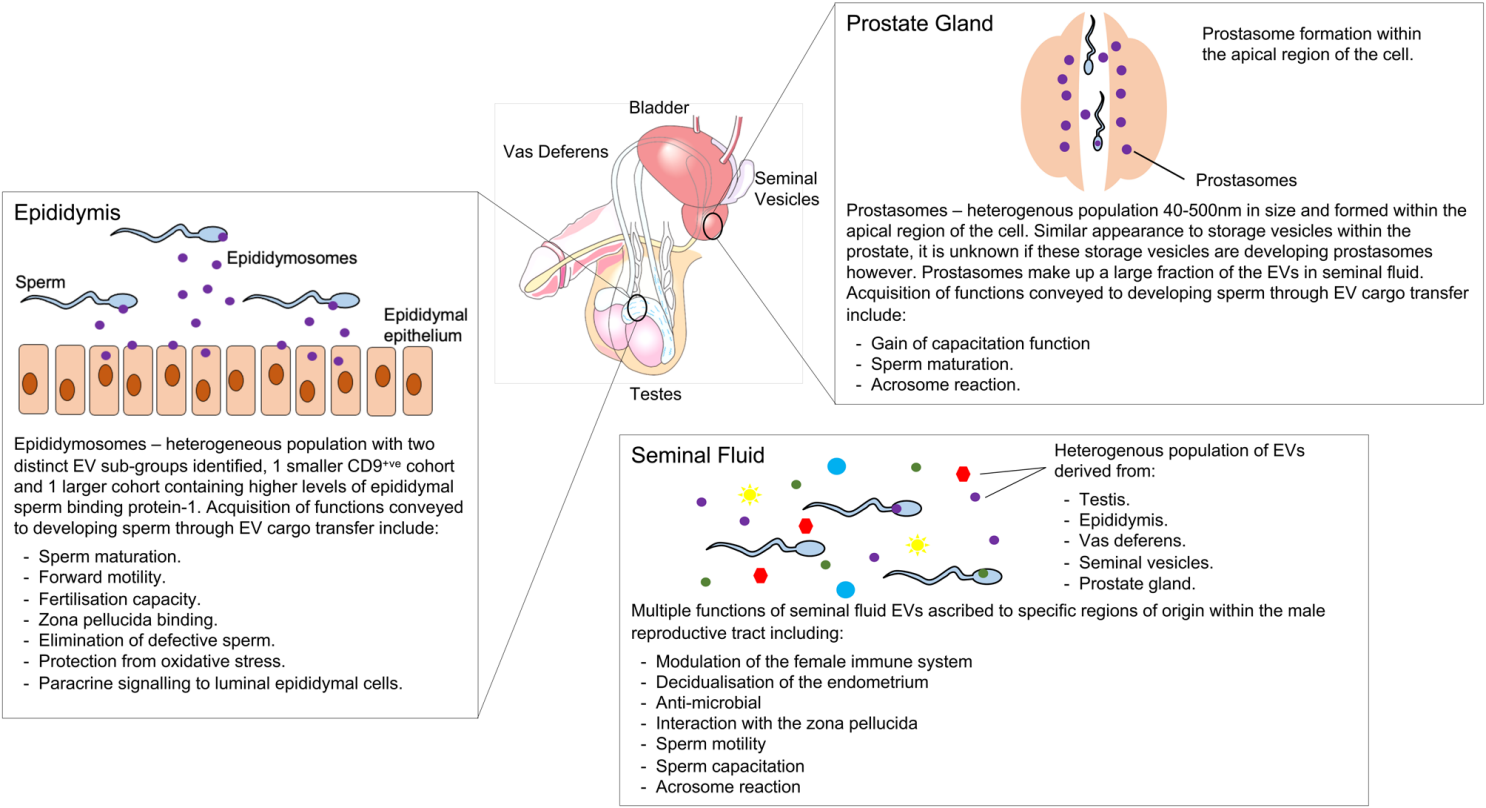
Extracellular vesicles from the male reproductive tract and their known functions.

Despite the extensive and comprehensive characterisation of EVs and their various functions from the epididymis and prostate, other regions of the male reproductive tract have yet to be studied extensively. Herein we discuss the provisional identification of EVs within the vas deferens, testes and the seminal vesicles, outlined in Fig. 4.

### Vas deferens-derived EVs

Following passage through the epididymis, sperm transitions to the vas deferens, which transports sperm stored within the epididymis to the ejaculatory duct and mixes with prostatic and seminal vesicle secretions (Sullivan *et al.* 2005). Secretions within the epididymis appear to have a role in modifying the environment of sperm cells prior to mixing with the prostate. Little is known about the function of EVs from the vas deferens but it may be possible that their release from this portion of the reproductive tract is limited, although the widespread release of EVs from other tissues suggests that this is unlikely (Manin *et al.* 1995). However, the mere identification of EVs from the vas deferens is insufficient to conclude that they interact with or modify sperm in any meaningful way.

### The testes

The release of EVs from within the testis, although highly probable given the heterogenous nature of EVs identified within seminal fluid, has yet to be identified in humans. However, the presence of EVs within the seminiferous tubule, the main site of sperm differentiation within the testis has been reported in turtles. Testis tissue from Chinese soft-shelled turtles, *Pelodiscus sinensis*, was examined using transmission electron microscopy and immunohistochemistry, identifying vesicle-like structures of a similar profile to exosomes, secreted into the seminiferous tubules (Ahmed *et al.* 2016). Further work exploring EVs released in the Chinese soft-shelled turtles was carried out using immunohistochemistry and immunofluorescence for the common EV marker CD63 and transmission electron microscopy for multivesicular bodies. Tarique *et al.* identified numerous CD63 signals within the basal compartment of the seminiferous tubules during early spermatogenesis, while during late spermatogenesis this was identified within the lumen of the seminiferous tubule. This recent finding suggests Sertoli cells within the testis secrete EVs into the seminiferous tubules during sperm differentiation. Given our understanding of the important roles, EVs play in other aspects of sperm development, it is highly plausible that these purported EVs may also play a crucial role in sperm development (Tarique *et al.* 2020).

### Seminal vesicles

The seminal vesicles are paired secretory glands adjacent to the prostate, lying on the base of the bladder, which secretes fluid rich in fructose and prostaglandins forming part of the ejaculate in which seminal fluid EVs are suspended. Analysis of the different fractions of the ejaculate, notably the prostatic fraction and seminal fluid fraction through centrifugation, revealed the presence of distinct EV markers. However, markers used to identify prostasomes (CD10, CD13 and CD26) were found to be absent within the seminal fluid pellet which instead contained heat shock protein-70 and CD55, both of which are known EV markers (Sahlén *et al.* 2010). Electron microscopy of prostate and seminal vesicle tissue demonstrated that EVs, unlike prostasomes, were released as single discrete structures by seminal vesicles rather than stored and secreted within larger secretory granules.

## Future perspectives

The field of EVs has grown rapidly in both an appreciation of their widespread distribution, identification in numerous body fluids and a greater understanding of their role in health and disease. The advent of technologies, not only to characterise EVs *in vivo* but also to isolate and study them *in vitro*, has led to significant advances in our understanding of their potential.

Identification of unique cargo within EVs offers new opportunities to develop novel diagnostic tools in order to recognise diseases that currently lack sensitive and specific tests. Increasing use of EVs both in science and healthcare, rapidly expanding cataloging of their cargo and falling costs of underlying technologies, will likely remove barriers to the routine use of EVs in healthcare settings as biomarkers to facilitate early disease detection and monitoring in the future.

A new and expanding area in EV research is their use as therapeutic targets, including delivery of therapeutics to previously hard-to-reach tissue. The ability to manipulate EVs either through the artificial incorporation of molecules such as short interfering RNA or modulating their interaction with cells represents a new potential field of clinical therapeutics.

Further work to understand how EVs are targeted or indeed how their surface ligands can be modified either physiologically or artificially represents a significant next step for the field of EV biology. This is of particular importance when characterising the release of EVs in the male reproductive tract, which compared to many other tissues, remains largely unstudied. The migration of primordial germ cells and development and maintenance of the germ cell niche during foetal development bears a striking resemblance to the development of cancer metastasis. A greater understanding of the development of this niche may give important insights as to how it can be preserved from damage and promote future fertility. The role of EVs in the development of a metastatic niche is well established, yet has never been studied in the development of the testicular stem cell niche. Although not in the male reproductive tract, EVs have been reported in the intestinal and haemopoietic stem cells niches (Laurenzana *et al.* 2018, Oszvald *et al.* 2020).

Currently, the release of EVs has not been reported in the testis in humans at different stages of development but has been extensively characterised in the epididymis and prostate, in the adult reproductive tract. Identification of EVs released in the foetal and pre-pubertal testis may give insights into the regulation of germ cell development and testicular pathologies across development, in a similar way to the modification of maturing sperm in the adult testis (Maciel & Mansuy 2019). Developments in the study of EVs released from the pre-pubertal male reproductive tract may lead to a greater understanding of how gonadotoxic exposures (e.g. chemotherapy) lead to lifelong damage to the testes. Furthermore, identification of EVs specific to the development of germ cell neoplasia *in situ* cells (GCNIS), the precursor for testicular germ cell tumours, may allow early diagnosis and treatment in the pre-malignant stage.

Although their release has only been investigated in the transport and storage components of the adult reproductive tract, EVs from these tissues have a clear and important role in the maturation and transformation of sperm. Failure of sperm to undergo this process and acquisition of new functions, mediated by EVs, leaves them incapable of performing their most essential biological function: traversing the female reproductive tract and fertilisation of an oocyte. Further modifications occur in the female reproductive tract including the adaptation of the female immune response to sperm and preparation of the endometrium for embryo impanation, not covered in this review.

Increasing knowledge regarding EVs is likely to provide exciting opportunities in the future for the development of EV-based diagnostic and therapeutic options. The potential role of EVs as novel vectors for drug delivery has recently been explored with numerous applications in a range of tissues (de Jong *et al.* 2019). Their use within the reproductive tract to deliver cargoes has only recently been explored. EVs generated from human embryonic kidney cells have been fluorescently labelled and visualised entering boar sperm. Crucially, no detrimental impact on sperm motility, viability and membrane integrity was identified which highlights their potential novel use to deliver cargoes to sperm (Vilanova-Perez *et al.* 2020). The interaction between sperm and an oocyte may then allow such cargoes to be incorporated into the developing embryo. Given recent developments in our understanding as to how paternal stressors alter EV cargoes with transcriptomic alterations in the offspring (Chan *et al.* 2020), we anticipate this will become an exciting and rapidly growing area of reproductive medicine.

The understanding of EVs in gamete and embryo development has grown in recent years as EVs identified within follicular fluid have been shown to interact with the developing conceptus (Gervasi *et al.* 2020, Han *et al.* 2020). This EV-mediated process not only includes communication within the same organism but also between paternal EVs and the maternal reproductive tract (Machtinger *et al.* 2016, Rodriguez-Caro *et al.* 2019). Delivery of EV cargo to the conceptus has been shown to enhance its development and has led some to speculate their lack of inclusion in current artificial reproductive technology (ART) may in part explain the poor development of some embryos in culture. Specific cargoes delivered to EVs include melatonin which was found to regulate reactive oxygen species in a rabbit model (Qu *et al.* 2020), while in bovine models follicular fluid EVs trigger granulosa cell proliferation (Hung *et al.* 2017), a finding also demonstrated in horses (da Silveira *et al.* 2012). Identification of the cargoes which induce these changes may lead to the development of bespoke ART media supplementation to improve the development of embryos in culture.

Despite the numerous advances in our understanding of EVs in the adult male reproductive tract, there is an urgent need for a greater understanding of the roles EVs play in the developing male reproductive tract (Baskaran *et al.* 2020). Based on the emerging evidence of EVs in sperm maturation and development, there seems little doubt that locally produced EVs will prove to play significant roles.

## Declaration of interest

The authors declare that there is no conflict of interest that could be perceived as prejudicing the impartiality of this review.

## Funding

M P R and R T M are funded by a MRC Centre for Reproductive Health Grant No: MR/N022556/1. R T M is funded by a UK Research and Innovation (UKRI) Future Leaders Fellowship MR/S017151/1.

## Author contribution statement

M P R conceived the idea for the article, undertook the literature search, wrote the manuscript, developed the figures and approved the manuscript for submission. C D G and R T M conceived the idea for the manuscript, undertook editorial changes and approved it for submission.
